# Risk Factors and Vaccination Dose Associated with COVID-19 Mortality: A Population-Based Study in Gyeongsangbuk-do, South Korea

**DOI:** 10.3390/pathogens15070721

**Published:** 2026-07-08

**Authors:** Na Young Hong, Minyu Qin, Min A Lim, Youkyoung Kim, Sook Hee Park, Sung Jun Park, Hyun Jun Kang, Byeong Ryeon Kim, Ji Hyuk Park, Seok Ju Yoo, Kwan Lee

**Affiliations:** 1 Department of Preventive Medicine, College of Medicine, Dongguk University, Gyeongju 38066, Republic of Korea; hny0989@naver.com (N.Y.H.); qmydgu77@gmail.com (M.Q.); minadid@korea.kr (M.A.L.); gbcidc6@gbcidc.or.kr (H.J.K.); skeyd@dongguk.ac.kr (J.H.P.); medhippo@dongguk.ac.kr (S.J.Y.); 2 Gyeongbuk Center for Infectious Diseases Control and Prevention, Andong 36759, Republic of Korea; gbcidc4@gbcidc.or.kr (S.H.P.); juncongo1@dongguk.ac.kr (S.J.P.); gbcidc8@gbcidc.or.kr (B.R.K.); 3 Division of Public Medicine, Gyeongsangbuk-do Provincial Government, Andong 36759, Republic of Korea; dbruddl9975@korea.kr

**Keywords:** COVID-19, SARS-CoV-2, risk factors, mortality, booster vaccination, older adults

## Abstract

Vaccination remains an effective intervention for reducing coronavirus disease 2019-related deaths, while the population-level evidence regarding the association between vaccination dose and mortality remains limited. This study aimed to investigate the association between vaccination status, independent risk factors, and COVID-19 mortality, using population-based surveillance data from Gyeongsangbuk-do, South Korea. A population-based retrospective cohort study was conducted using 698,537 confirmed cases from Gyeongsangbuk-do, between January 2021 and June 2022, including 1008 deaths. Univariable and multivariable logistic regression analyses were performed to identify factors associated with mortality. Additional analyses were conducted among adults aged ≥ 65 years. The overall case fatality rate was 0.14%, increasing to 0.88% among older adults. Older age (≥65 years; OR = 87.262, 95% CI: 67.265–114.472) and underlying disease (OR = 20.394, 95% CI: 17.136–24.270) were strongly associated with mortality. After adjustment for sex, age, and underlying disease, unvaccinated individuals had substantially higher odds of death than vaccinated individuals (aOR = 7.897, 95% CI: 6.903–9.034). Booster vaccination (≥3 doses) was associated with markedly reduced mortality in both the overall population (aOR = 0.094, 95% CI: 0.081–0.109) and older adults (aOR = 0.119, 95% CI: 0.102–0.139). Advanced age, underlying diseases, and lack of vaccination were significant factors associated with COVID-19 mortality during the mass vaccination period. These findings support continued efforts to improve booster vaccination coverage among older adults and individuals with underlying diseases.

## 1. Introduction

Coronavirus disease 2019 (COVID-19) continues to impose a substantial burden on global public health systems, resulting in considerable morbidity, mortality, and pressure on healthcare services worldwide. Older adults and individuals with underlying diseases are particularly vulnerable to severe illness and death after infection [1,2]. South Korea experienced several large-scale outbreaks during the mass vaccination period, despite the implementation of nationwide vaccination programs.

Vaccination remains a critical public health strategy for reducing severe infection, hospitalization, and mortality associated with COVID-19 [3]. However, accumulating evidence suggests that vaccine-induced immunity wanes over time [4]. This decline appears to be more pronounced among older adults [5,6]. Consequently, booster vaccination strategies were widely implemented to maintain immune protection. Increasing evidence demonstrates that the COVID-19 vaccination plays an essential role in reducing severe illness and mortality [7,8]. Booster vaccination further enhances vaccine effectiveness against severe COVID-19 outcomes [9]. However, the real-world population-level evidence of the association between vaccination dose status and COVID-19 mortality remains limited.

Gyeongsangbuk-do is located in the southeastern part of South Korea and comprises a diverse mix of urban and rural communities. As one of the most rapidly aging regions in the country, it provides an important setting for investigating population-level risk factors associated with COVID-19 mortality.

This study aimed to investigate factors associated with COVID-19 mortality and to evaluate the association between vaccination status, particularly booster vaccination, and mortality risk, using population-based surveillance data from Gyeongsangbuk-do, South Korea, during the mass vaccination period.

## 2. Materials and Methods

### 2.1. Data Collection and Study Design

**Figure 1:**
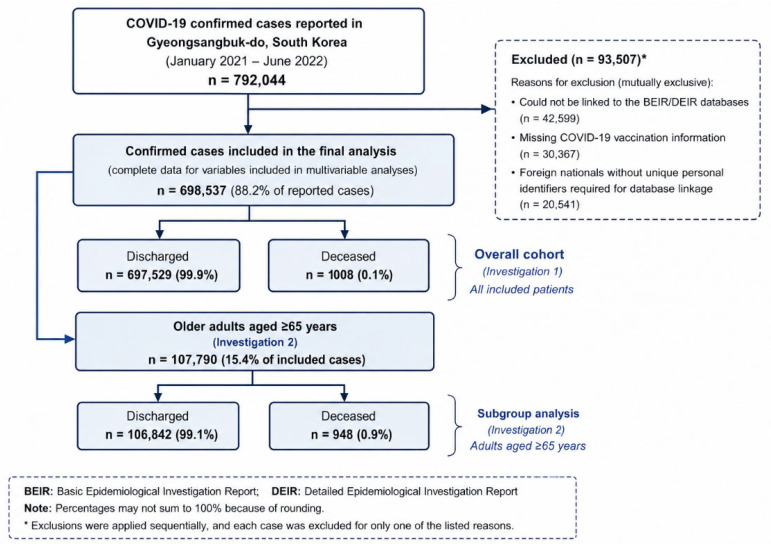
Flow diagram of participant selection.

The study initially identified 792,044 confirmed COVID-19 cases reported in Gyeongsangbuk-do, South Korea, between January 2021 and June 2022, including 1048 deaths. Cases were excluded if they could not be linked to the Basic Epidemiological Investigation Report (BEIR) and Detailed Epidemiological Investigation Report (DEIR) databases, lacked COVID-19 vaccination information, or were foreign nationals without the unique personal identifiers required for linkage. After these exclusions, 698,537 confirmed cases, including 1008 deaths, with complete information for the variables included in the multivariable analyses were included in the final analysis. The participant selection process is shown in Figure 1.

First, all eligible cases were included to investigate risk factors associated with COVID-19 mortality (Investigation 1). Subgroup analyses were then performed according to age, sex, and comorbidity status to evaluate whether these associations differed across demographic and clinical characteristics. Vaccination status, including the number of vaccine doses received, was included as an independent variable to assess its association with COVID-19 mortality. Finally, individuals aged ≥ 65 years were analyzed separately to identify mortality risk factors among older adults (Investigation 2). This subgroup comprised 107,790 individuals, including 948 deaths, because older adults were at particularly high risk for severe COVID-19 outcomes and accounted for more than 90% of all COVID-19-related deaths during the study period.

### 2.2. Study Variables

Demographic characteristics (age and sex), vaccination status, and underlying disease status were obtained from the epidemiological investigation reports. Underlying diseases were analyzed as a composite variable (yes/no) because detailed information on individual comorbid conditions was not consistently available.

Although height and weight information was available in the surveillance database, these variables contained substantial missingness and implausible values, likely reflecting inconsistencies in measurement units or data entry. Therefore, BMI and obesity were not included in the composite underlying disease variable or the multivariable analyses to avoid potential misclassification and selection bias.

### 2.3. Statistical Analysis

Confirmed COVID-19 cases (including both recovered and deceased patients) are presented as frequencies and percentages. Inter-group differences were assessed using Pearson’s chi-squared test. Multivariable logistic regression analysis was performed to identify factors associated with COVID-19 mortality. Variables included in the multivariable models were selected a priori based on clinical relevance and previous literature and included sex, age group, underlying disease status, and vaccination status. Multicollinearity among the independent variables was assessed using the variance inflation factor (VIF), and all VIF values were below 2, indicating no evidence of substantial multicollinearity. Interaction terms were not evaluated because the primary objective of this study was to estimate the independent associations between predefined risk factors and COVID-19 mortality using a prespecified multivariable model. A *p*-value less than 0.05 was considered statistically significant. Statistical analyses and visualization were performed using R software (version 4.5.3).

### 2.4. Ethics

Ethical approval of this study was granted by the Institutional Review Board of Dongguk University Gyeongju Hospital (IRB No: 110757-202512-HR-02-02).

## 3. Results

**Figure 2:**
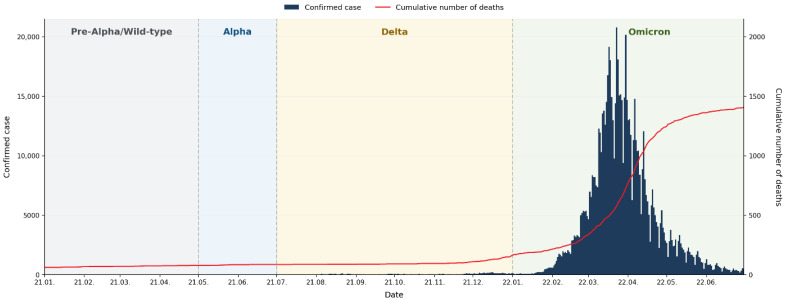
Confirmed cases and cumulative number of deaths in Gyeongsangbuk-do, January 2021–June 2022.

During the study period, the overall mortality rate in Gyeongsangbuk-do was 61.6 per 100,000 population. Beginning in February 2022, confirmed cases surged, accompanied by a rapid increase in the cumulative number of deaths (Figure 2).

We analyzed individuals from all age groups (Table 1 and Table 2). Table 1 presents data for the entire cohort, showing that 1008 of 698,537 individuals (0.144%) were deceased. Women accounted for 56.2% of deceased cases, which is a proportion significantly higher than that of men (*p* = 0.037). Moreover, 94.1% of deceased cases were aged 65 or older (*p* < 0.001). Consistent with this, 85.2% of deceased individuals had underlying diseases (*p* < 0.001), and unvaccinated individuals represented 44.1% of fatalities (*p* < 0.001). Among discharged cases, 45.1% had received ≥3 doses, while 26.4% were unvaccinated.

Table 2 highlights significant associations between mortality and age, underlying diseases, and vaccination status. The proportion of deceased cases increased markedly with age. Among individuals aged ≥ 65 years, the case fatality rate was 0.88%. Notably, 74.2% of deceased cases were individuals aged ≥ 80 years, and 85.9% had underlying diseases. Unvaccinated individuals accounted for 43.4% of deaths in the older group. The findings differed from those observed in the overall study population, with no statistically significant difference according to sex. The distribution of baseline characteristics, including underlying disease prevalence and COVID-19 vaccination status according to age group, is presented in Supplementary Table S1.

Logistic regression analysis emphasized the elevated odds for older adults aged ≥ 65 years (OR = 87.262, 95% CI: 67.265–114.472) and individuals with underlying diseases (OR = 20.394, 95% CI: 17.136–24.270) (Table 3). The extremely high odds ratio observed among individuals aged ≥ 65 years likely reflects the markedly concentrated mortality burden in older adults during the study period, as more than 90% of COVID-19-related deaths occurred in this age group. Similar age-associated increases in mortality risk have been consistently reported in previous population-based COVID-19 studies.

We further performed logistic regression analysis specifically within the older population subgroup. While underlying disease remained significantly associated with mortality, the magnitude of this association was lower than that observed in the overall population. Vaccination was associated with lower odds of death, with ≥3 doses showing an OR of 0.400 (95% CI: 0.346–0.463) compared with unvaccinated individuals.

**Figure 3:**
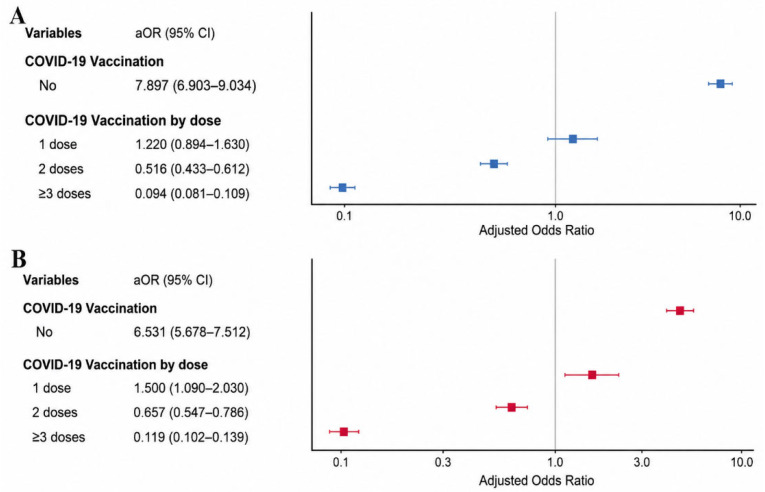
Adjusted odds ratio and forest plot. Adjusted by sex, age, and underlying disease. Panel (**A**): aOR for participants across all age groups. Panel (**B**): aOR for participants aged ≥ 65 years.

To minimize the potential confounding effects of sex, age, and underlying comorbidities on the observed association between vaccination and mortality, adjusted odds ratios (aORs) for vaccination status and vaccination dose are presented in Figure 3. After adjustment for confounding factors, unvaccinated individuals had substantially higher odds of death than vaccinated individuals in both the overall population (aOR = 7.897, 95% CI: 6.903–9.034) and older adults (aOR = 6.531, 95% CI: 5.678–7.512). Individuals who received more than two vaccine doses showed significantly lower odds of death compared with the unvaccinated. The protective association was particularly significant in those who received the three-dose vaccine in all groups (aOR (all ages) = 0.094, 95% CI: 0.081–0.109; aOR (aged ≥ 65 years) = 0.119, 95% CI: 0.102–0.139).

## 4. Discussion

The analysis using population-based surveillance data from Gyeongsangbuk-do, South Korea, revealed that older age and underlying disease were notably associated with COVID-19 mortality during the mass vaccination period. The observed reduction in mortality risk among the vaccinated population is consistent with a protective association of vaccination, particularly the booster vaccination (≥3 doses).

Our study shows that full vaccination was associated with significantly lower mortality across all ages, aligning with global evidence on vaccine effectiveness [10]. A study on COVID-19 mortality risk among adults with chronic kidney disease indicated that vaccination effectively prevents symptomatic infection and reduces mortality rates [11,12]. Similarly, full vaccination is associated with lower rates of severe illness, shorter hospital stays, and reduced mortality [13]. After adjustment, the odds of death were over six times higher among unvaccinated individuals than among vaccinated individuals. A Hungarian study similarly demonstrated that vaccination status reduced all-cause mortality compared to no vaccination, after adjusting for measured confounders and potential vaccinator effects [14].

Notably, the protective association was more pronounced among participants who received the booster vaccination, corresponding to 88.1–90.6% lower odds of death. Although the standard two-dose regimen was also associated with lower odds of death (34.3–48.4% lower odds of death), individuals fully vaccinated with only two doses retained significantly higher odds of death compared with those who received a booster vaccination (≥3 doses).

Interestingly, a single vaccine dose was not associated with a statistically significant reduction in mortality after adjustment for potential confounders. This finding may reflect the limited immune protection achieved shortly after receipt of a single vaccine dose, as adequate humoral immunity generally requires additional time and subsequent vaccine doses to develop. In addition, some individuals may have been infected shortly after receiving a single vaccine dose, before an adequate immune response had developed, which could have attenuated the apparent protective effect of a single dose. Furthermore, individuals who received only a single vaccine dose may have represented a selected subgroup with a higher baseline risk of mortality, including older adults and those with underlying medical conditions who were prioritized during the early phase of the COVID-19 vaccination program. Therefore, the apparent lack of protection associated with a single dose should not be interpreted as evidence of vaccine ineffectiveness, but rather as reflecting incomplete immunization and residual confounding inherent to observational studies.

The delayed development and heterogeneity of antibody responses to a single vaccine dose could result from the time interval required to mount sufficient humoral immunity, which becomes more robust after consecutive doses [15,16].

Additionally, the slightly higher apparent risk in this group might be due to the infections that happened soon after the initial dose. A significant elevation of antibodies was observed after the second dose of vaccination [17]. Nevertheless, the two-dose vaccination strategy might not provide optimal and sustained protection. A test-negative case–control study of COVID-19-related hospitalization revealed that vaccine effectiveness against symptomatic COVID-19 declined within five months after two doses, especially among immunosenescent individuals (the elderly and those with clinical risk conditions) [18]. Therefore, booster vaccination is crucial for preserving long-term defense against serious consequences and COVID-19-related death [19]. Previous studies have shown that booster vaccination significantly increases neutralizing antibody responses and offers longer-lasting immune protection [20]. Interestingly, the rate of antibody decline appears to be slower after booster vaccination, indicating sustained protective efficacy.

Higher expression of the SARS-CoV-2 receptor has been associated with advanced age and comorbidities and may contribute to increased disease severity [21]. Age is a critical factor affecting the time required for SARS-CoV-2 clearance [22,23]. Experiments in rhesus macaques also showed that older animals exhibit more interstitial pneumonia and viral replication than younger ones [24]. In our study, individuals aged ≥ 65 years showed a strong association with mortality (OR = 87.262, 95% CI: 67.265–114.472), particularly those aged 80 and above (OR = 14.281, 95% CI: 10.899–18.713). This trend may be explained by immunosenescence, the aging-related decline of innate immune cells, including T cells and B cells [25,26]. Immunosenescence involves immune dysfunction associated with mitochondrial impairment [27], delayed virus detection, and impaired antigen presentation, all of which could hinder viral clearance. A prospective study of SARS-CoV-2 cases in acute care hospitals suggested a higher initial viral load in the elderly is likely linked to mortality [28]. Another proposed mechanism is the development of a prothrombotic state in older adults [29], which may promote systemic inflammation and thrombotic complications, thereby increasing the risk of mortality, particularly in individuals with underlying medical conditions.

In addition, older adults have a substantially higher prevalence of chronic underlying diseases, which may further increase vulnerability to severe COVID-19 outcomes. Although underlying diseases were adjusted for in the multivariable model, the exceptionally large association observed for advanced age should not be interpreted as being solely attributable to age-related immune dysfunction, but rather as reflecting the combined effects of immunosenescence and the greater burden of comorbidities among older adults. To further assess the robustness of this finding, we additionally performed a sensitivity analysis in which age was modeled as a continuous variable. The association remained statistically significant, with each one-year increase in age associated with a 12.2% increase in the odds of COVID-19 mortality (adjusted OR, 1.122; 95% CI, 1.115–1.129), indicating that the observed association was robust regardless of whether age was modeled as a categorical or continuous variable.

Comorbidities are a significant risk factor for COVID-19 mortality. Individuals with comorbidities had approximately 2- to 20-fold increased odds of mortality in our study. We found that 85.2% of deceased cases had comorbidities, with hypertension (40.5%), diabetes (22.0%), and cardiovascular disease (13.2%) being the most common. A retrospective study by Djaharuddin I et al. reported similar findings among hospitalized patients, with hypertension (42.31%), cardiovascular disease (30.77%), and diabetes (28.21%) being the most common comorbidities [30]. Moreover, more than half of the patients with COVID-19 in that study had two or more comorbidities. Impaired innate immune function in patients with comorbidities and adverse drug reactions may negatively affect the expression of SARS-CoV-2 entry factors and hinder the production of infection-fighting antibodies [31,32]. The reduced ability to mount a coordinated immune response warrants particular attention in patients with comorbidities [33].

As a sensitivity analysis, calendar period was additionally included in the multivariable model. The association between vaccination status and COVID-19 mortality remained statistically significant after adjustment for calendar period (adjusted OR, 7.83; 95% CI, 6.83–8.97), indicating that the observed association was robust to temporal variation during the study period. Nevertheless, residual confounding may still remain because several important clinical and epidemiological factors, including changes in circulating SARS-CoV-2 variants, vaccination coverage, and clinical management, were unavailable in the surveillance database.

Some limitations should be considered when interpreting the findings of this study. First, causal relationships between vaccination status and COVID-19 mortality could not be definitively established because of the retrospective observational design. Second, several potentially important confounding factors were not available in the surveillance database, including smoking status, time since vaccination, previous SARS-CoV-2 infection, reinfection, hybrid immunity, circulating SARS-CoV-2 variants, clinical severity at presentation (e.g., pneumonia, intensive care unit admission, or mechanical ventilation), the interval from symptom onset to diagnosis, socioeconomic status, healthcare accessibility, and therapeutic interventions. Therefore, these variables could not be included in the multivariable analyses, and residual confounding cannot be excluded. In particular, because the number of vaccine doses and the recency of vaccination are closely correlated, individuals who received a booster dose generally did so more recently than those who received only the primary vaccination series. Therefore, the observed association between a greater number of vaccine doses and lower mortality may partly reflect differences in the recency of vaccination, and consequently less waning of vaccine-induced immunity, rather than the cumulative number of doses alone. Because individual vaccination dates were not available in the surveillance database, the effect of vaccine dose could not be distinguished from that of time since the most recent vaccination. Therefore, the dose–response findings should be interpreted with caution. Furthermore, healthy vaccinee (healthy user) bias cannot be excluded. Individuals who received vaccination, particularly booster doses, may have differed systematically from unvaccinated individuals in healthcare-seeking behavior, functional status, frailty, socioeconomic characteristics, and other unmeasured factors independently associated with mortality. Although we adjusted for age, sex, and underlying disease, these behavioral and socioeconomic factors could not be accounted for. Therefore, the observed protective association between vaccination and COVID-19 mortality may partly reflect healthy vaccinee bias rather than a purely causal protective effect. In addition, detailed information on individual comorbid conditions was not available; therefore, comorbidity was analyzed as a composite variable rather than disease-specific categories. Nevertheless, the overall burden of comorbidity may better reflect the clinical vulnerability of patients with COVID-19, particularly among older adults with multiple chronic conditions. Mortality may also have been influenced by temporal changes during the study period, including changes in the predominant SARS-CoV-2 variants, vaccination coverage, and clinical management, which could not be fully accounted for in the present analyses. Additionally, because this study was based on confirmed cases, asymptomatic infections may have been underreported, potentially leading to overestimation of mortality rates. Therefore, the findings should be interpreted cautiously, and further longitudinal studies incorporating more comprehensive clinical, behavioral, and socioeconomic data are warranted.

## 5. Conclusions

This large-scale population-based study suggests that advanced age, underlying comorbidities, and lack of vaccination were associated with an increased risk of COVID-19 mortality. While the standard two-dose regimen was associated with lower odds of death, booster vaccination (≥3 doses) showed a more pronounced association with reduced mortality, with 88.1–90.6% lower odds of death compared with unvaccinated individuals.

These findings underscore the critical importance of strategies for maintaining long-term protection, particularly among vulnerable populations, and reducing the burden of COVID-19-related deaths.

## Figures and Tables

**Table 1:** General characteristics of COVID-19 confirmed cases for all age groups (Unit: %).

Variables	Categories	Total (n = 698,537)	Confirmed Cases		*p* Value
			Discharged (n = 697,529)	Deceased (n = 1008)	
Sex	Man	47.8	47.8	43.8	0.037
	Woman	52.2	52.2	56.2	
Age group (years)	<65	84.6	84.7	6.9	<0.001
	≥65	15.4	15.3	94.1	
Underlying disease	Yes	22.1	22.0	85.2	<0.001
	No	77.9	78.0	14.8	
	Hypertension (yes)	11.1	11.1	40.5	<0.001
	Diabetes mellitus (yes)	5.6	5.6	22.0	<0.001
	Dyslipidemia (yes)	4.4	4.4	5.8	0.038
	Cardiovascular disease (yes)	1.6	1.5	13.2	<0.001
	Cerebrovascular disease (yes)	1.0	1.0	12.7	<0.001
	Cancer (yes)	0.9	0.9	5.0	<0.001
	Chronic obstructive pulmonary disease (yes)	0.2	0.2	5.5	<0.001
	Pneumonia (yes)	0.1	0.1	5.8	<0.001
	Chronic kidney disease (yes)	0.5	0.5	6.3	<0.001
	Psychiatric disorder (yes)	0.6	0.6	7.2	<0.001
	Tuberculosis (yes)	0.1	0.1	0.6	<0.001
	Asthma or allergic disease (yes)	1.2	1.2	2.4	<0.001
	Immunocompromised condition (yes)	0.35	0.35	2.3	<0.001
COVID-19 Vaccination dose *	None	21.5	26.4	44.1	<0.001
	1 dose	7.4	2.5	7.1	
	2 doses	26.0	26.0	18.7	
	≥3 doses	45.1	45.1	30.1	

* Percentages for vaccination dose were calculated among all confirmed cases.

**Table 2:** Demographic and clinical characteristics of older adults with COVID-19 (Unit: %).

Variables	Categories	Total (n = 107,790)	Confirmed Cases		*p* Value
			Discharged (n = 106,842)	Deceased (n = 948)	
Sex	Man	40.8	40.8	41.7	0.850
	Woman	59.2	59.2	58.3	
Age (years)	65–69	32.7	33.0	6.0	<0.001
	70–74	23.1	23.2	10.1	
	75–79	15.3	15.3	9.7	
	≥80	28.9	28.5	74.2	
Underlying diseases	Yes	67.4	67.3	85.9	<0.001
	No	32.6	32.7	14.1	
COVID-19 Vaccination dose *	None	12.6	12.3	43.4	<0.001
	1 dose	3.3	3.3	7.1	
	2 doses	8.1	8.0	18.7	
	≥3 doses	75.4	75.8	30.6	

* Percentages for vaccination dose were calculated among all confirmed cases.

**Table 3:** Univariable logistic regression analysis of mortality risk.

Variables	Categories	All Age Groups	Age ≥ 65
		OR * (95% CI)	OR (95% CI)
Sex	Man	ref	-
	Woman	1.177 (1.039–1.333)	-
Age group (years)	<65	ref	-
	≥65	87.262 (67.265–114.472)	-
Age group (years)	65–69	-	ref
	70–74	-	2.396 (1.726–3.327)
	75–79	-	3.469 (2.492–4.829)
	≥80	-	14.281 (10.899–18.713)
Underlying disease	No	ref	ref
	Yes	20.394 (17.136–24.270)	2.953 (2.459–3.546)
COVID-19 Vaccination	Yes	ref	ref
	No	2.484 (2.181–2.831)	9.010 (7.855–10.335)
COVID-19 Vaccination by dose	None	ref	ref
	1 dose	1.903 (1.476–2.454)	0.633 (0.488–0.822)
	2 doses	0.430 (0.363–0.510)	0.693 (0.580–0.828)
	≥3 doses	0.400 (0.346–0.463)	0.119 (0.103–0.139)

* Age was categorized as <65 and ≥65 years in Investigation 1 and as 65–69, 70–74, 75–79, and ≥80 years in Investigation 2.

## Data Availability

The data used in this work are available from the corresponding author upon reasonable request.

## Supplementary Materials

The following supporting information can be downloaded at: https://www.mdpi.com/article/10.3390/pathogens15070721/s1. Supplementary Table S1: Baseline characteristics according to age group.
