# Supplementary material for: Risk Factors and Vaccination Dose Associated with COVID-19 Mortality: A Population-Based Study in Gyeongsangbuk-do, South Korea
Source: Pathogens. 2026 Jul 8;15(7):721. doi: 10.3390/pathogens15070721 (PMC13414630; doi:10.3390/pathogens15070721)
Supplement: Supplementary file 1 [file pathogens-15-00721-s001.zip › pathogens-4378648-supplementary.pdf]

**Supplementary Table S1. Baseline characteristics according to age group.**

| Variables | Categories | Total (N=698,537) | <65 (n=543,950) | ≥65 (n=154,587) | P value |
|---|---|---|---|---|---|
| Underlying disease (yes) | | 22.1 | 13.9 | 67.4 | < 0.001 |
| Specific comorbidities | Hypertension (yes) | 11.1 | 5.6 | 41.4 | < 0.001 |
| | Diabetes mellitus (yes) | 5.6 | 3.0 | 19.9 | < 0.001 |
| | Dyslipidemia (yes) | 4.4 | 2.9 | 12.7 | < 0.001 |
| | Cardiovascular disease (yes) | 1.6 | 0.6 | 6.8 | < 0.001 |
| | Cerebrovascular disease (yes) | 1.0 | 0.4 | 4.7 | < 0.001 |
| | Cancer (yes) | 0.9 | 0.6 | 2.3 | < 0.001 |
| | Chronic obstructive pulmonary disease (yes) | 0.2 | 0.1 | 1.0 | < 0.001 |
| | Pneumonia (yes) | 0.1 | 0.1 | 0.5 | < 0.001 |
| | Chronic kidney disease (yes) | 0.5 | 0.3 | 1.6 | < 0.001 |
| | Psychiatric disorder (yes) | 0.6 | 0.4 | 2.0 | < 0.001 |
| | Tuberculosis (yes) | 0.1 | 0.1 | 0.2 | < 0.001 |
| | Asthma or allergic disease (yes) | 1.2 | 1.1 | 1.8 | < 0.001 |
| | Immunocompromised condition (yes) | 0.4 | 0.3 | 0.8 | < 0.001 |
| COVID-19 Vaccination (Yes) | | 23.9 | 7.0 | 21.3 | < 0.001 |
| | 1 dose | 72.4 | 70.2 | 84.3 | <0.001 |
| | 2 doses | 69.8 | 67.3 | 83.4 | <0.001 |
| | ≥3 doses | 47.3 | 41.7 | 78.0 | <0.001 |

*Percentages for vaccination dose were calculated among vaccinated individuals only.
